# Treatment variables associated with outcome in emergency department patients with suspected sepsis

**DOI:** 10.1186/s13613-020-00747-8

**Published:** 2020-10-14

**Authors:** Narani Sivayoham, Lesley A. Blake, Shafi E. Tharimoopantavida, Saad Chughtai, Adil N. Hussain, Andrew Rhodes

**Affiliations:** 1 Department of Emergency Medicine, St George’s University Hospitals NHS Foundation Trust, Blackshaw Road, London, SW17 0QT UK; 2 Department of Anaesthesia and Intensive Care Medicine, St George’s University Hospitals NHS FT and St George’s University of London, Cramner Terrace, Tooting, London, SW17 0RE UK

**Keywords:** Sepsis, Septic shock, Emergency department, Blood pressure, Time-to-treatment, Antibiotics

## Abstract

**Background:**

Early treatment is advocated in the management of patients with suspected sepsis in the emergency department (ED). We sought to understand the association between the ED treatments and outcome in patients admitted with suspected sepsis. The treatments studied were: (i) the time to antibiotics, where time zero is the time the patient was booked in which is also the triage time; (ii) the volume of intravenous fluid (IVF); (iii) mean arterial pressure (MAP) after 2000 ml of IVF and (iv) the final MAP in the ED.

**Methods:**

We performed a retrospective analysis of the ED database of patients aged ≥ 18 year who met two SIRS criteria or one red flag sepsis criteria on arrival, received intravenous antibiotics for a suspected infection and admitted between 8th February 2016 and 31st August 2017. The primary outcome measure was all-cause in-hospital mortality. The four treatments stated above were controlled for severity of illness and subject to multivariate logistic regression and Cox proportional-hazard regression to identify independent predictors of mortality.

**Results:**

Of the 2,066 patients studied 272 (13.2%) died in hospital. The median time to antibiotics was 48 (interquartile range 30–82) minutes. The time to antibiotics was an independent predictor of mortality only in those who developed refractory hypotension (RH); antibiotics administered more than 55 mins after arrival was associated with an odds ratio (OR) for mortality of 2.75 [95% confidence interval (CI) 1.22–6.14]. The number-needed-to-treat was 4. IVF > 2000 ml (95% CI > 500– > 2100), except in RH, and a MAP ≤ 66 mmHg after 2000 ml of IVF were also independent predictors of mortality. The OR for mortality of IVF > 2,000 ml in non-RH was 1.80 (95% CI 1.15–2.82); Number-needed-to-harm was 14. The OR for morality for a MAP ≤ 66 mmHg after 2000 ml of IVF was 3.42 (95% CI 2.10–5.57). A final MAP < 75 mmHg in the ED was associated with, but not an independent predictor of mortality. An initial systolic blood pressure of < 100 mmHg has a sensitivity of 63.3% and specificity of 88.4% for the development of RH.

**Conclusion:**

In this study, antibiotics were found to be time-critical in RH. Intravenous fluids > 2000 ml (except in RH) and a MAP ≤ 66 mmHg after 2000 ml of IVF were also independent predictors of mortality.

## Background

Early treatment is advocated to reduce mortality in sepsis [1–4]. The emergency department (ED) has a critical role in the identification and treatment of sepsis as the majority of patients in hospital with sepsis are admitted as emergencies [5, 6]. It is important to ensure that those treatments are delivered in a timely and optimal manner to improve outcome whilst minimising harm. Four early treatments are advocated in the management of sepsis: early antibiotics, intravenous fluids, early diagnosis of refractory hypotension (RH) and early commencement of vasopressors for RH to achieve a minimum mean arterial pressure (MAP) of 65 mmHg [1, 2].

Antibiotics are crucial to the management of sepsis. The relationship between the timing of antibiotic delivery and outcome is controversial. Some studies support early antibiotics, whereas others have found no impact on mortality [7–10]. In some studies where a significant proportion of the study population had refractory hypotension, early antibiotics were found to be associated with a reduction in mortality [7, 8]. Attempting to deliver early antibiotics in suspected sepsis has led to antibiotics being delivered to patients who do not have a final diagnosis of an infection and risks the development of antibiotic resistance. We have found that approximately 9% of patients receiving early antibiotics in the ED did not have a final diagnosis of an infection [11]. Whilst this number will never reach zero, it is important to minimise the delivery of unnecessary antibiotics. It is therefore important to identify the group of patients in whom early antibiotics makes a difference to outcome.

Intravenous fluids (IVF) are important to maintain circulating volume in sepsis, but the volume of IVF is controversial [12] as some argue it may be harmful [13]. Trials are underway to determine the optimal fluid regime in sepsis [14, 15].

A low MAP after a bolus of intravenous fluids in known to be associated with increased mortality. The Sepsis-3 [16] definition of refractory hypotension (RH) is the need for vasopressors to maintain a MAP > 65 mmHg after an adequate fluid bolus. When the lactate measured after the fluid bolus is over 2 mmol/l, the RH is termed septic shock [17]. Shankar-Hari et al. [17] performed a systematic review and meta-analysis of 44 studies with varying definitions of shock. The criteria for defining refractory hypotension in the definition above was set at a MAP of 65 mmHg and agreed to by a panel of experts. In adults, the generally accepted volume of fluid before determining if a patient has RH is 2000 ml [18], representing 30 ml/kg in the average 70 kg adult. As the cut-off point for defining RH was specified and not derived, we felt the precise cut-off point for prediction of mortality should be studied.

We have previously reported that achieving a final MAP of 65 mmHg or more before leaving the ED was an independent treatment variable associated with survival [19] in a group of patients with sepsis and refractory hypotension or a lactate of ≥ 4 mmol/l or both.

## Study objectives

  (i) To understand the association between the time to antibiotics and in-hospital mortality in the following groups of patients:
    - All patients,
    - Those with RH,
    - Those with a REDS score of 5–12, a group who have a mortality rate (MR) similar to those with RH.
  (ii) To determine the critical time interval from arrival for the delivery of antibiotics for the patient groups in whom the timing of antibiotics was associated with mortality.
  (iii) To identify patients on arrival in the ED in whom the timing of antibiotics is time-critical.
To understand the association between the volume of IVF administered in the ED and mortality.
To determine the MAP after 2000 ml of IVF that is associated with mortality.
To understand the association between the final MAP in the ED and mortality.

## Methods

### Study design, time period and setting

This is a retrospective study of prospectively collected data. In this study, we combined the derivation and validation population we used to derive and validate the Risk-stratification of ED suspected Sepsis (REDS) score [11]. During the study period there was an expectation, guided by the Sepsis CQUIN (Commissioning for Quality and Innovation) [20] that patients presenting with red flag sepsis [3] receive antibiotics within an hour of arrival. This expectation has since been revised to the delivery of antibiotics within an hour of diagnosis rather than an hour from arrival. The ED is also expected to discharge or transfer 95% of patients within 4 h of arrival. The antibiotics that were delivered were in accordance with the local hospital guidelines for empiric antibiotics.

### Participant selection and measurements

Adults who received intravenous antibiotics for the treatment of suspected sepsis and were admitted, were studied. In addition to the previously collected data, the urea, creatinine, the volume of fluid (crystalloids or blood) commenced (infusions of ≤ 100 ml were not included) in the ED and the time of delivery of the antibiotics were noted. The time to delivery of antibiotics was calculated from the time of registration (this also represents the triage time) in the ED and administration of antibiotics. A history of hypertension was also collected. The systolic blood pressure (SBP) and diastolic blood pressure (DBP) after completing 2000 ml of intravenous fluids and final blood pressure (BP) in the ED were noted and the MAP was calculated from these readings. The collected data were checked by a second researcher for accuracy. The REDS score was calculated for each patient [11].

Exclusions: the following patients were excluded: (i) patients who did not have the time of antibiotic administration documented and (ii) those who did not have a blood pressure recorded after the initial blood pressure recording.

### Outcome measure

The primary end-point was all-cause in-hospital mortality.

### Data analysis

Univariate analyses were carried out between survivors and non-survivors for the following treatment and patient variables: the time to antibiotics, the volume of intravenous fluid commenced in the ED, the final MAP and the REDS score. In those who received a minimum of 2000 ml of IVF who had a BP recorded after completion of the second litre of fluid, the MAP after the second litre was also studied.

The following analyses were carried out:

- To determine if a treatment variable was an independent predictor of mortality, multivariate logistic regression (MVLR) and Cox proportional-hazard regression (CPHR) were carried out on the four treatment variables stated above, urea, creatinine and the REDS score. The latter three patient variables were used to control for the severity of illness. The populations studied were: (a) all patients, (b) those with RH, and (c) those who had a BP measured after 2000 ml of IVF, and (d) those with a REDS score of 5–12.
- For treatment variables that were found to be independent predictors of mortality, a receiver operator characteristic (ROC) curve was constructed and the cut-off point identified. The optimum cut-off point was determined by the statistical software package. The OR for mortality was calculated for this cut-off point as was the actual risk reduction (ARR) or actual risk increase (ARI); the numbers-needed-to-treat (NNT) or harm (NNH) were calculated, respectively.
- If patients with RH were found to benefit from time-critical administration of antibiotics, then it is important to identify them on arrival. RH is not diagnosed until a minimum 2000 ml of fluid are administered. Therefore, a patient may be identified as requiring time-critical antibiotics after the critical time has elapsed. Initial SBP, DBP, MAP, respiratory rate, heart rate and the shock index (HR/SBP) were subject to univariate analysis for the development of RH. Those variables that were found to be significant on univariate analysis for the development of RH had ROC curves constructed and cut-off points identified. Sensitivities and specificities for other more practical cut-off points were also studied. In order to limit the number of antibiotics delivered to those in whom it does not make a difference to outcome, cut-off points with greater specificity than sensitivity for mortality, were chosen. In addition, to limit the number of patients who are false negative to this initial criterion, we speculated on a safety net.
- The final MAP, a treatment end-point, and the MAP after 2000 ml of IVF was studied further by constructing a ROC curve for mortality and identifying the cut-off point. The MR was calculated for these two treatment variables, divided in to 5 mmHg bands. The MR associated with the final MAP was also calculated for those with and without a history of hypertension.

### Statistics

MedCalc Statistical Software version 18.1 (MedCalc Software, Ostend, Belgium) was used for statistical analysis. Statistical significance was defined as *p* < 0.05. Univariate analysis was performed using the Mann–Whitney test where data were not normally distributed. The Chi-square test was used to determine the significance of differences in mortality between groups. The odds ratio (OR) was calculated to determine the scale of the difference in mortality between two groups. The area under the ROC (AUROC) curve was measured and was deemed significant if it and the 95% confidence interval (95% CI) were > 0.5. MVLR and the Cox proportional hazards regression models were constructed using the ‘Enter’ method. Variables were entered if *p* < 0.05 and removed if *p* > 0.1. For the MVLR model treatment variables that are known to be significantly associated with outcome were included even if the univariate analysis was not significant [21, 22]. Discrimination and calibration of the MVLR models were assessed by the AUROC curve and the Hosmer–Lemeshow test [23], respectively.

### Sample size and missing data

Steill et al. [24] advocate a minimum ten outcomes (deaths) per variable. This study had a maximum four treatment variables (time to antibiotics, volume of fluid, MAP after 2000 ml of fluid and the final MAP) and a maximum of three patient variables, namely the REDS score, urea and creatinine. The number of variables studied in any population or sub-group should have a minimum of ten deaths per variable studied. Patients who were missing data for the REDS score were not excluded.

## Results

The baseline characteristics of the study population can be found in Table 1 with further details in our previous publication [11].

**Table 1:** Baseline characteristics of study population

Variable	Median [interquartile range] or number (percentage); total 2066 patients
Age	71 [54–82] years
Sex (male)	1,035 (50.1%)
Malignancy	413 (20%)
Refractory hypotension	117 (5.7%)
Admission to the intensive care unit	253 (12.2%)
Mortality	272 (13.2%)
Hospital length of stay (days)	6 [3–13] days

**Fig. 1:**
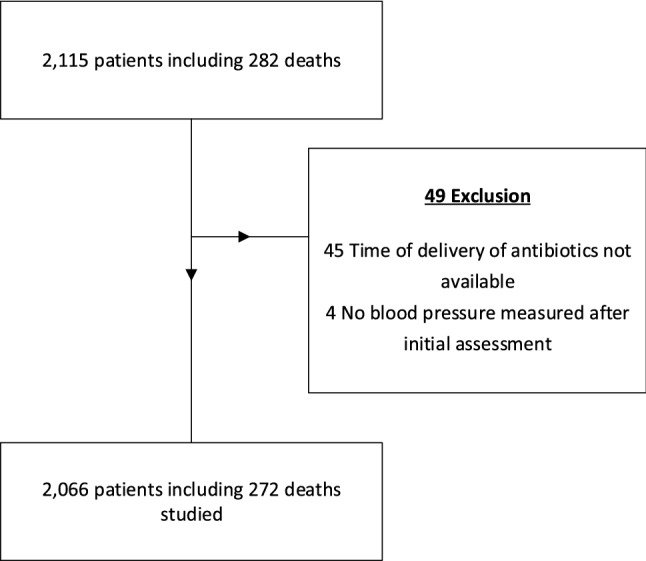
Patient flow diagram

Exclusions can be found in Fig. 1. Of the 2,066 patients studied, 1800 (87.1%) met the red-flag criteria [3] for antibiotics within an hour. Of the 2,066 patients studied, 1600 (77.4%) arrived by ambulance; 1047 (50.7%) arrived by priority ambulance. Of the 272 non-survivors, 251 (92.2%) arrived by ambulance, 190 (70%) arrived as priority ambulance. Seventeen patients received blood; 9 received one unit and 8 received two units of blood. Each unit of blood was estimated to be 250 ml. None of the continuous variables in this study were normally distributed.

**Fig. 2:**
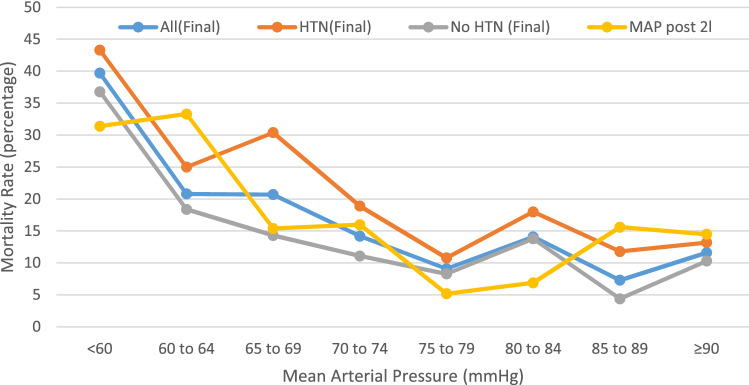
Mortality rates associated with the final mean arterial pressure (MAP) and the MAP after 2000 ml of intravenous fluid in the ED. *ED *emergency department, *l *litre

Results as per study objectives:

*(i) To understand the association between the time to antibiotics and in-hospital mortality:*
  - *The whole population.*
    Table 2 illustrates the median times and interquartile ranges for the time to antibiotics. Antibiotics were delivered within 60 min in 1328 (64.3%) patients and within 240 min in 98.1% of the study population; 39 received antibiotics after 240 min from registration, the longest taking 446 min.
    Non-survivors received their antibiotics significantly sooner (median 44 min) than survivors (median 49 min, *p* = 0.011), Table 2. Non-survivors also had significantly higher REDS scores than survivors; a median 4 in non-survivors compared to a median of 2 in survivors. On MVLR against the REDS score, to control for severity of illness, the timing of antibiotics was not an independent predictor of mortality, *p* = 0.89. The finding of the MVLR model was confirmed by CPHR analysis.
    Of the 1,328 patients who received antibiotics within an hour 183 (13.8%) died, compared to 89 (12.1%) deaths amongst the 738 patients who received antibiotics after 60 min from arrival. There was no difference in mortality, *p* = 0.28.
  - *Patients with refractory hypotension*
    Univariate analysis of the time to antibiotics in 117 patients with RH (Table 3) shows no difference between survivors and non-survivors. But MVLR against the REDS score, to control for severity of illness, revealed the time to antibiotics to be an independent predictor of mortality (*p* = 0.03) and confirmed by CPHR (*p* = 0.022).
  - *Patients with a REDS score of 5–12*
    The MR in those with RH was 37.6% was similar to the 34.8% MR in patients with REDS scores of 5–12. MVLR analysis of the 328 patients with REDS scores of 5–12, found the time to antibiotics was not associated with mortality, *p* = 0.93.
    (ii)To determine the critical time to antibiotics
    The time to antibiotics was an independent predictor of mortality only in patients who were found to have RH. A ROC curve was constructed for the time to antibiotics, for mortality. The AUROC curve was 0.6 (95% CI 0.502–0.69) and the cut-off point for association with mortality was at > 55 min (95% CI 17–65). Of the 80 patients who received antibiotics at or within 55 min from arrival, 24 died (MR 30%) compared to 20 deaths amongst the 37 (MR 54.1%) who received antibiotics after 55 min (range 56–249 mins), p = 0.015. The OR for mortality if antibiotics were delivered > 55 min after arrival was 2.75 (95% CI 1.22–6.14). The absolute risk reduction was 24.1% and the NNT was 4.
    (iii) Identification of patients on arrival, in whom antibiotics is time critical
    Initial SBP, DBP, MAP and SI were significantly different in those who developed RH compared to those who did not develop RH. This was not the case for heart rate and respiratory rate. The AUROC for the development of RH was highest for initial SBP and MAP. Although the AUROC for initial SBP and initial MAP were identical we chose to study initial SBP further as this would be the most familiar at initial assessment. The cut-off point for the association of initial SBP to RH was a SBP ≤ 107 mmHg. As this is not a practical number, the test characteristics for an initial SBP of < 110 mmHg and < 100 mmHg were studied. The sensitivity and specificity for a cut-off point of < 110 mmHg for initial SBP was 75.2% and 76.7%, respectively. Similarly, a cut-off point of < 100 mmHg had a sensitivity and specificity of 63.3% and 88.4%, respectively. If an initial SBP < 100 mmHg were to be implemented as the new criteria for early antibiotics, only 301 patients would be deemed high-risk requiring antibiotics within an hour of arrival compared to 1,800 patients who met the red-flag criteria [3]. Patients who meet red-flag criteria are expected to receive antibiotics within an hour of meeting that criteria.
    The speculated safety net for those who are had a SBP of 100 mmHg or more on arrival but became hypotensive during their ED stay, would be to deliver antibiotics within 30mins of a drop in SBP to < 100 mmHg.
*To understand the association between the volume of IVF administered in the ED and mortality*
  In the study population as a whole, the volume of fluid was not significantly different between survivors and non-survivors but when controlled for severity of illness by the REDS score, it was an independent predictor (Table 2) of mortality. The AUROC curve was 0.53 (95% CI 0.51–0.55) with a cut-off point of > 2000 ml (95% CI > 500– > 2100). Of the 219 patients who received > 2000 ml of intravenous fluid 52 died (MR 23.7%) compared to 220 death amongst the 1,847 patients (MR 11.9%) who received ≤ 2000 ml of intravenous fluids, *p* < 0.0001. Amongst the 117 patients with RH, the mortality rate in those who received > 2000 ml was 32.5% compared to 46.2% in those who received ≤ 2000 ml, *p* = 0.22. MVLR and CPHR of the treatment variables showed that the volume of IVF was not an independent predictor of mortality in those with RH (Table 3). Similar analysis on the 1949 without RH showed that the volume of fluid was an independent predictor of mortality on MVLR (*p* = 0.004) and CPHR (*p* = 0.01). Of the 1,949 patients without RH there were 26 deaths amongst the 141 patients (MR 18.4%) who received > 2000 ml of fluid compared to 202 deaths amongst the 1,808 patients (MR 11.2%) who received ≤ 2000 ml, *p* = 0.014. The OR for mortality for IVF > 2000 ml in non-RH is 1.80(95% CI 1.15–2.82). The number needed to harm was 14.
*To determine the MAP after 2000 ml of fluid that is associated with mortality*
  Of the 674 patients who received a minimum 2000 ml of IVF, a BP after completion of 2000 ml was recorded in 503 patients (Table 4). MVLR of the MAP after 2000 ml of IVF against the REDS score, revealed that the MAP after 2000 ml IVF was an independent predictor of mortality, *p* = 0.02 (Table 4). The ROC curve of the MAP after 2000 ml of IVF identified a cut-off point of ≤ 66 mmHg for association with mortality; the MR for a MAP ≤ 66 mmHg was 31.3% and 13.3% for a MAP > 66 mmHg. The OR for mortality after 2,000 ml of IVF for a MAP ≤ 66 mmHg was 3.42 (95% CI 2.10–5.57). The MRs associated with the MAP, divided in to 5 mmHg bands, after 2000 ml of fluid are illustrated in Fig. 2.
*To determine to the optimal final blood pressure in the ED*

**Table 2:** Univariate and multivariate logistic regression and Cox proportional hazard regression of patient and treatment variables for all patients

Treatment variable	All *n* = 2066 median (IQR)	Survivors *n* = 1794 median (IQR)	Non-survivors *n *= 272 median (IQR)	Univariate analysis	OR (95% CI) on MVLR	Significance of OR on MVLR	Cox proportional hazard ratio (95%CI)	Significance of Cox proportional hazard ratio
Time to antibiotics (mins)	48 (30–82)	49 (31–83)	44 (25–77.5)	*p* = 0.011^a^	1.00 (0.99–1.00)	*p* = 0.89	1.00 (0.99–1.00)	*p* = 0.97
Volume of IVF (ml)	1000 (1000–2000)	1000 (1000–2000)	1000 (1000–2000)	*p *= 0.1	0.99 (0.99–0.99)	*p* = 0.0001^a^	0.99 (0.99–0.99)	*p* = 0.0006^a^
Final MAP (mmHg)	83 (74–93)	84 (75–93)	81 (69–91)	*p* < 0.0001	1.01 (0.99–1.02)	*p* = 0.55	1.00 (0.99–1.01)	*p* = 0.62
Urea (mmol/l)	6.7 (4.6–10.8)	6.3 (4.4–9.5)	12.1 (6.9–19.9)	*p* < 0.0001^a^	1.07 (1.05–1.09)	*p* < 0.0001^a^	1.03 (1.02–1.04)	*p* < 0.0001^a^
Creatinine (micromol/l)	89 (67–125)	86 (67–117)	117 (76–192)	*p* < 0.0001^a^	0.99(0.99–1.00)	p = 0.06	0.99(0.99–1.00)	*p* = 0.51
REDS score	3 (2–4)	2 (2–3)	4 (3–6)	*p* < 0.0001^a^	1.67 (1.55–1.80)	*p* < 0.0001^a^	1.35 (1.27–1.43)	*p* < 0.0001^a^

Hosmer–Lemeshow test for multivariate logistic regression *p* = 0.28

*IVF* intravenous fluid, *CI* confidence interval, *MAP* mean arterial pressure, *REDS score* Risk-stratification of Emergency Department suspected Sepsis score, *IQR* interquartile range, *OR* Odds Ratio, *MVLR* multivariate logistic regression

^a^Statistical significance reached

**Table 3:** Univariate and multivariate logistic regression and Cox proportional-hazard regression analyses of patient and treatment variables in patients with refractory hypotension (RH)

Treatment variable	All *n* = 117 median (IQR)	Survivors *n* = 73 median (IQR)	Non-survivors *n* = 44 median (IQR)	Univariate analysis	OR (95% CI) on MVLR	Significance of OR on MVLR	Cox proportional hazard (95%CI)	Significance of Cox proportional hazard
Time to antibiotics (mins)	37 (20–62)	33 (19–53)	46 (22.5–78.5)	*p* = 0.08	1.01 (1.001–1.02)	*p* = 0.03^a^	1.01 (1.001–1.012)	*p* = 0.022^a^
Volume of IVF (ml)	3000 (2000–3870)	3000 (2000–3903)	2800 (2000–3750)	*p* = 0.26	0.99 (0.99–1.00)	*p* = 0.10	0.99 (0.99–1.00)	*p* = 0.067
Final MAP (mmHg)	61 (56–68)	62 (56–71)	59.5 (55–65)	*p* = 0.12	0.97 (0.93–1.01)	*p* = 0.14	0.99 (0.96–1.02)	*p* = 0.45
REDS score	7 (6–9)	7 (5–8)	8 (7–10.5)	*p* = 0.001	1.53 (1.23–1.92)	*p* = 0.0002^a^	1.37 (1.17 – 1.60)	*p* = 0.0001^a^

Hosmer–Lemeshow test for multivariate logistic regression *p* = 0.24

*IVF * intravenous fluid, *CI* confidence interval, *MAP* mean arterial pressure, *REDS score* Risk-stratification of Emergency Department suspected Sepsis score, *IQR* interquartile range, *OR* odds ratio, *MVLR* multivariate logistic regression

^a^Statistical significance reached

**Table 4:** Univariate and multivariate logistic regression and Cox proportional hazard regression analyses of the treatments and the REDS score of patients who had a blood pressure measured after a minimum 2000 ml of intravenous fluids

Treatment variable	All *N* = 503 median (IQR)	Survivors *N* = 419 median (IQR)	Non-survivors *N *= 84 median (IQR)	Univariate analysis	OR (95% CI) on MVLR	Significance of OR on MVLR	Cox proportional hazard ratio (95% CI)	Significance of Cox proportional hazard ratio
Time to antibiotics (mins)	39 (24–62)	39 (24–62)	38.5 (22–67.5)	*p* = 0.71	1.00 (0.99–1.01)	*p* = 0.25	1.00 (0.99–1.01)	*p* = 0.28
Volume of IVF (ml)	2000 (2000–3000)	2000 (2000–3000)	2625 (2000–3000)	*p* = 0.0003^a^	1.00 (0.99–1.00)	*p* = 0.77	1.00 (0.99–1.00)	*p* = 0.64
MAP mmHg post 2 l fluid bolus	77 (66–90)	78 (68–90)	69 (60–88)	*p* = 0.0008^a^	1.03 (1.00–1.05)	*p* = 0.02^a^	1.03 (1.00–1.05)	*p* = 0.02^a^
Final MAP (mmHg)	79 (69–89)	80 (71–89)	70.5 (60–82.5)	*p* < 0.0001^a^	0.99 (0.96–1.02)	*p* = 0.5	0.99 (0.97–1.02)	*p* = 0.5
Urea (mmol/l)	7.7(4.8–13.8)	7(4.5–11.7)	15.5(9–26)	*p* < 0.0001^a^	1.05 (1.03–1.08)	*p* < 0.0001^a^	1.03 (1.01–1.04)	*p* = 0.003^a^
REDS score	4 (2–6)	3 (2–5)	6 (5–8)	*p* < 0.0001^a^	1.63 (1.42–1.87)	*p* < 0.0001^a^	1.43 (1.29–1.58)	*p* < 0.0001^a^

Hosmer–Lemeshow test for multivariate logistic regression; *p* = 0.84

*CI* confidence interval, *MAP* mean arterial pressure, *REDS score* Risk-stratification of Emergency Department suspected Sepsis score *IQR* interquartile range, *OR* odds ratio, *MVLR* multivariate logistic regression

^a^Statistical significance reached

Univariate analysis of the final MAP for the study population as a whole is shown in Table 2. The ROC curve of final MAP shows a cut-off point of ≤ 73 (95% CI ≤ 69– ≤ 84) mmHg to be associated with mortality. However, on MVLR and CPHR the final MAP was not an independent predictor of mortality when controlled for severity of illness as per the REDS score. The REDS score includes initial and refractory hypotension. Rounding up this cut-off point for the final MAP to the nearest 5 mmHg showed the following: a final MAP < 75 mmHg was found in 494 patients of whom 100 died, a MR of 20.2%. Of the 1572 who had a final MAP ≥ 75 mmHg 172 died, a MR of 10.9%, *p* < 0.0001. Figure 2 illustrates the crude mortality rates associated with the final MAP divided in to bands of 5 mmHg. The graph illustrates the whole study population and the population divided in to those with and without a history of hypertension. A final MAP of ≥ 75 mmHg was associated with a lower mortality rate irrespective of a history of hypertension.

## Discussion

In our study we have explored the impact of the ED treatment variables on outcome in patients admitted with suspected sepsis. We found the time to antibiotics to be an independent predictor of mortality in those who developed RH and to have a cut-off point at 55 min from arrival. The NNT for the delivery of antibiotics within 55 min of arrival, was 4. Therefore, in cases of refractory hypotension, for every four patients, the timely delivery of antibiotics saved one life. A volume > 2000 ml of IVF was found to be an independent predictor of mortality except in those with RH. A MAP ≤ 66 mmHg after 2000 ml of fluid was also found to be an independent predictor of mortality. A final MAP of ≥ 75 mmHg was associated with a lower mortality rate compared to a final MAP of < 75 mmHg but not an independent predictor of mortality.

The median time to antibiotics was 48 min and over 98% of patients received antibiotics within four hours of registering in the ED. The time to antibiotics was identified as critical in RH. The initial vital sign that would identify those who are likely to go in to RH was an initial SBP of ≤ 107 mmHg. As this was not a rounded number an initial SBP < 100 mmHg, was studied. An initial SBP < 100 mmHg had a sensitivity of 63.3% and a specificity of 88.4% for the identification of RH. The use of an initial SBP of < 100 mmHg to deliver antibiotics within an hour may reduce the number of patients receiving early and unnecessary antibiotics. However, the reduction in sensitivity for RH may be mitigated by a safety net to deliver antibiotics within 30 mins of reaching a SBP < 100 mmHg, should the SBP drop below 100 mmHg after arrival in the ED. Furthermore, we did not find the time to antibiotics to be time critical in those with a REDS score of 5 or more, a group with a mortality rate similar to those with RH.

In a multi-centre study, Seymour et al. [8] studied over 49,000 ED patients who had the sepsis 3 h- treatment bundle commenced within six hours of arrival in the ED. The population studied were those with severe sepsis [25] and septic shock. This latter group is now known as refractory hypotension. Seymour et al. [8] studied only those with organ dysfunction which is a sub-group of the ED population with suspected sepsis. They found the risk adjusted mortality rate for the time to antibiotics increased by an OR 1.04 for each hour delay in the administration of antibiotics. But 45% of the population studied had septic shock compared to the 5.7% satisfying the RH criteria in our study population. The findings by Seymour et al. [8] may be explained by the heavy weighting towards septic shock patients in their study population. The hourly odds ratio for mortality in relation to antibiotic delivery is available for those on and not on vasopressors (Seymour et al. [8] Supplementary file Figure S3) but not for those with and without shock. The study population categorises 22,336 patients as having septic shock although only 16,721 received vasopressors. The OR for mortality with each hour delay in antibiotic delivery was significant only in those receiving vasopressors and not significant in the 32,610 patients who did not receive vasopressors. Alam et al. [9] performed a multi-centre, open label, randomised controlled trial of prehospital antibiotics in sepsis. The median time to antibiotics in the ED was 70 mins and the median time to delivering antibiotics in the pre-hospital setting was 26 mins before arrival in the ED; a median 96 min between the two arms of the study. This study found that giving antibiotics in the prehospital setting did not improve outcome. Only 3.7% of this population had septic shock, a population similar to ours. These two studies concur with our finding that the timing of antibiotic delivery is critical only in those with refractory hypotension but not in the general ED population suspected of having sepsis.

We did not find the volume of fluid to be an independent predictor of mortality in those who developed RH. In fact, in this group, those who received over 2000 ml of fluid have a lower mortality rate, although this did not reach statistical significance. A clearer view could be formed when the current on-going trials [14, 15] report their findings. However, at volumes of > 2000 ml, we found intravenous fluids to be an independent predictor of mortality in those without RH, with a number-needed to harm of 14. This finding has not been reported before and would need external validation. Lane et al. [26] studied the association between prehospital of intravenous fluids and in-hospital mortality in patients with sepsis. They found prehospital fluids were associated with a reduction in mortality [OR 0.73 (95% CI 0.56–0.95)] in those with hypotension but not in those without hypotension. In the latter group, there was a trend towards increased mortality with prehospital fluids but this did not reach statistical significance [OR 1.41 (95% CI 0.81–2.44)]. These findings of pre-hospital fluid administration being associated with reduced mortality in those with hypotension and a trend towards increased mortality in those without hypotension, is in keeping with our study findings. As our study is a retrospective observational study, it could be argued that there may be other confounders that have not been studied may have influenced the outcome. We have tried to mitigate this by controlling the volume of fluid against the other conditions that would demand increased fluids such at the renal function and final blood pressure. However, we acknowledge that there may be other unknown factors that may have influenced the result.

Our study identified that a MAP ≤ 66 mmHg after 2000 ml of IVF was an independent predictor of mortality despite regressing it against the REDS score which includes RH. This suggests the current definition of RH, the need for vasopressors after a fluid bolus to maintain a MAP of ≥ 65 mmHg, may not be sufficient. In addition, we found a final of MAP of ≥ 75 mmHg was associated with a lower mortality rate but it was not an independent predictor or mortality. Our finding is supported by the findings by Moman et al. [27] who studied patients with and without acute kidney injury (AKI) in septic shock. Those without an AKI had a significantly higher median post-resuscitation MAP of 71 mmHg compared to a median MAP of 66 mmHg in those with an AKI. Asfar et al. [28] studied a target MAP 65–70 and 80–85 mmHg in septic shock and found no difference in mortality. But the group who should have their MAP titrated to 65–70 mmHg were actually treated to a higher MAP. These results suggest that a target MAP of ≥ 75 mmHg may be better than a MAP of ≥ 65 mmHg. However, as the final MAP was not an independent predictor of mortality in our retrospective observational study, the association with reduced mortality may be a result of a variable that we have not studied. The question whether a target MAP of ≥ 75 mmHg is better than a target MAP of ≥ 65 mmHg could be resolved by a randomised controlled trial, thus eliminating any unknown confounders.

Our study has several limitations. Firstly, it is a single centre study. Secondly, the number of patients with RH is small. Thirdly, it is a retrospective observational studies. Such studies are not ideal for the assessment of the impact of treatments as factors that influence the outcome may not have been studied. Fourthly, the appropriateness of antibiotics was not studied but it is intuitive that this may have an impact on outcome. Finally, we did not study the other treatment such as intubation and commencement of vasopressors that may have influenced outcome.

## Conclusion

In this retrospective single centre study, the time to antibiotics was critical only in those who subsequently developed refractory hypotension; the cut-off point for the delivery of antibiotics was 55 min and was associated with an NNT of 4. Intravenous fluid volumes > 2000 ml (except in refractory hypotension) was an independent predictor of mortality with a number-needed-to-harm of 14. A MAP ≤ 66 mmHg after 2000 ml of intravenous fluid was also found to be independent predictors of mortality with an OR for mortality of 3.42 (95% CI 2.10–5.57). A final MAP of < 75 mmHg in the ED was associated with but not an independent predictor of mortality.

## Data Availability

All data used are included in the manuscript.
